# Case Report: Clip migration induced recurrent choledocholithiasis after laparoscopic common bile duct exploration

**DOI:** 10.3389/fmed.2026.1869401

**Published:** 2026-06-03

**Authors:** Bin Zhou, Yingchao Lu, Peng Chang, Hongxing Xu, Danfeng Shen

**Affiliations:** 1 Department of General Surgery, Suzhou TCM Hospital Affiliated to Nanjing University of Chinese Medicine, Suzhou, China; 2 Department of Hepatobiliary Surgery, Taicang Affiliated Hospital of Soochow University, Suzhou, China

**Keywords:** clip migration, Hem-o-lok clip, laparoscopic common bile duct exploration (LCBDE), metallic clip, recurrent choledocholithiasis, T-tube drainage

## Abstract

Clip migration into the common bile duct (CBD) represents a rare yet severe complication after laparoscopic biliary surgery. Herein, we report a case of a 75-year-old male patient who previously received simultaneous laparoscopic cholecystectomy (LC) and laparoscopic common bile duct exploration (LCBDE) with T-tube drainage. The patient was admitted with upper abdominal pain and jaundice. Elevated inflammatory indicators and liver function enzymes indicated biliary obstruction complicated by cholangitis. Abdominal computed tomography (CT) revealed CBD stones and two intraductal high-density foreign bodies, highly suggestive of migrated surgical clips. After empirical antibiotic administration, the patient underwent emergent reoperative LCBDE and T-tube reinsertion on hospital day 2. Intraoperative exploration confirmed complete clearance of biliary stones, with one Hem-o-lok clip and one titanium clip embedded in the stones. The patient had an uneventful postoperative course and was discharged with a favorable prognosis. This case report combined with literature review further clarifies the etiological characteristics of clip migration, and provides practical references for the early prevention and individualized management of such rare biliary complications.

## Introduction

At this stage, laparoscopic technology has become the mainstream approach for the treatment of biliary tract stones, which can significantly reduce surgical trauma and shorten hospital stay (1). For patients with choledocholithiasis who have undergone biliary surgery, severe consequences such as cholangitis, obstructive jaundice and pancreatitis caused by stone recurrence constitute a challenging postoperative complication. The recurrent choledocholithiasis is associated with various risk factors, including biliary anatomical factors (2), metabolic factors (3), and surgery-related factors (4), all of which may increase the risk of recurrence.

In addition, the migration of surgical clips used for ligating blood vessels or bile ducts into the CBD is a rare but long-term iatrogenic complication inducing stone recurrence and cholangitis (5, 6). The mechanism of clip migration is not yet fully clear, which may be related to local inflammation, inaccurate clipping, surgical clips being too close to the CBD, or postoperative bile leakage (7). Therefore, clip migration should be included in the differential diagnosis for patients with prior biliary surgery presenting with biliary symptoms (8). We report a case of simultaneous migration of a metal clip and a Hem-o-lok clip after LCBDE with T-tube drainage, which has never been reported previously. This study is reported in line with the SCARE criteria (9).

## Case presentation

A 75-year-old male patient presented to the emergency department with a one-month history of intermittent upper abdominal pain. Vital signs were stable upon admission. Physical examination showed mild cutaneous and scleral jaundice, along with significant upper abdominal tenderness. The patient had underlying hypertension and diabetes mellitus, with well-controlled blood pressure and blood glucose levels.

Sixteen months before admission, the patient underwent percutaneous transhepatic gallbladder drainage (PTGBD) for acute obstructive suppurative cholangitis (AOSC) complicated with cholecystolithiasis and choledocholithiasis. Two months after PTGBD, LC combined with LCBDE and T-tube drainage was performed, and the T-tube was electively removed at 6 weeks postoperatively. Review of surgical records confirmed the use of three Hem-o-lok clips and one titanium clip during the operation. Specifically, one Hem-o-lok clip and one titanium clip were applied to ligate the cystic artery, and two additional Hem-o-lok clips were used for closure of the cystic duct. Following T-tube placement, the CBD incision was sutured with 4–0 absorbable sutures (Vicryl, ETHICON).

**Figure 1:**
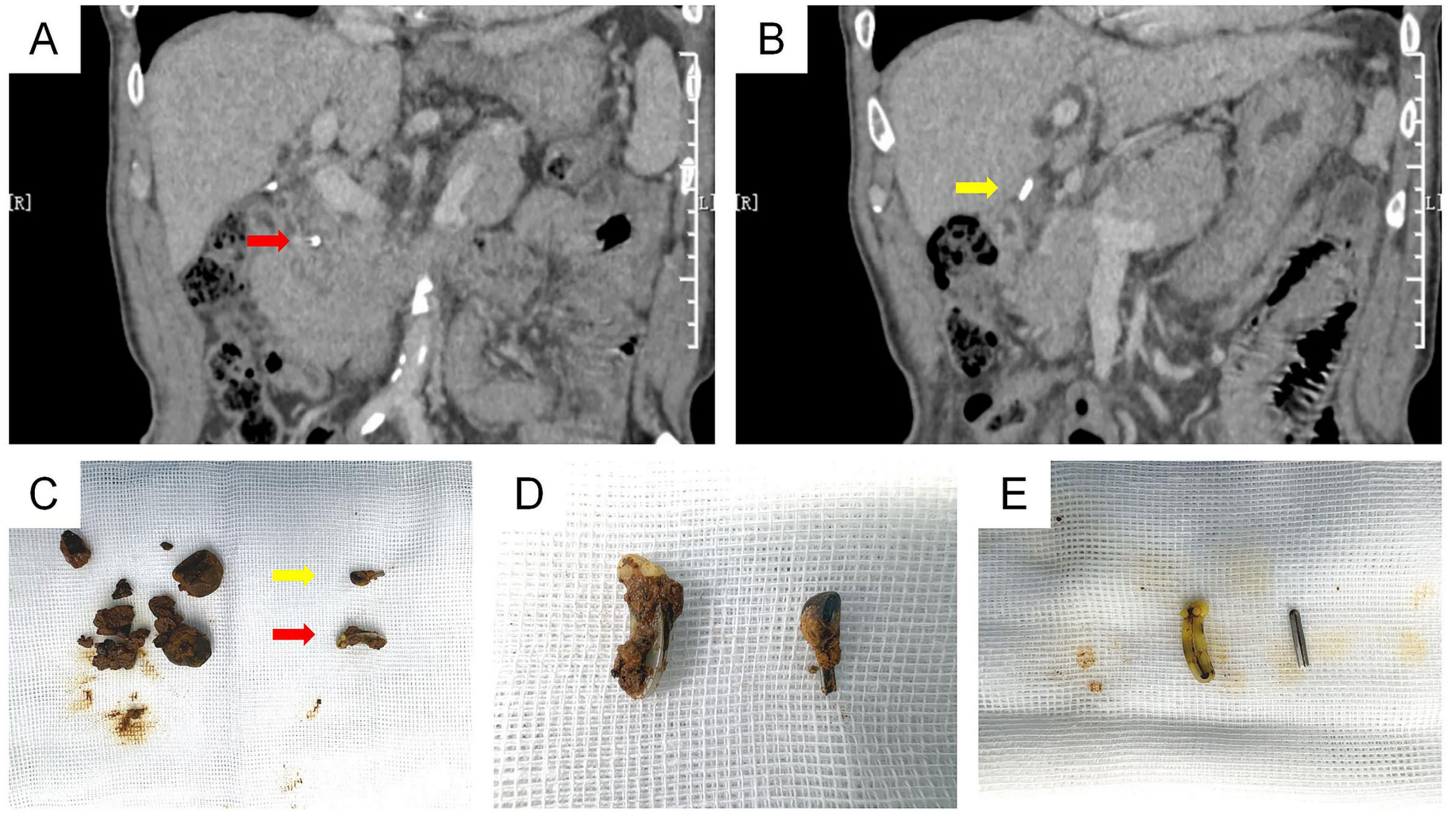
Preoperative CT findings and retrieved stone specimen. **(A,B)** Preoperative CT revealed biliary ductal dilation with multiple CBD stones and two high-density foreign bodies: a Hem-o-lok clip (red arrow) and a metallic clip (yellow arrow). **(C–E)** A Hem-o-lok clip (red arrow) and a metallic clip (yellow arrow) were embedded in the stones.

Laboratory examinations showed significantly elevated inflammatory and liver function indices: C-reactive protein (CRP) 121.8 mg/L (0.0–10.0 mg/L), procalcitonin (PCT) 3.22 ng/mL (0.0–0.05 ng/mL), total bilirubin (TB) 94.0 μmol/L (3.0–22.0 μmol/L), aspartate transaminase (AST) 64.0 U/L (17.0–59.0 U/L), alanine aminotransferase (ALT) 59.0 U/L (0.0–50.0 U/L), and *γ*-glutamyl transpeptidase (GGT) 187.0 U/L (15.0–73.0 U/L). The level of carbohydrate antigen 19–9 (CA 19–9) also increased markedly to 409.1 U/mL (0.0–37.0 U/mL). Emergency abdominal computed tomography (Revolution CT, GE Healthcare) revealed biliary ductal dilation with multiple CBD stones, together with two distinct high-density foreign bodies within the CBD (Figures 1A,B). Therefore, a diagnosis of clip migration complicated with recurrent choledocholithiasis was highly suspected.

The diagnosis was challenging due to nonspecific biliary obstructive symptoms and low sensitivity of ultrasonography for detecting migrated clips. Our diagnostic reasoning integrated the patient’s previous LCBDE history, recurrent abdominal pain, abnormal liver function, and imaging evidence of bile duct stones and residual clips. Differential diagnoses included primary choledocholithiasis, recurrent cholangitis, biliary stricture, postoperative bile duct injury, malignant biliary tract tumor, and residual stones from the initial surgery. After timely empirical antibiotic treatment, the patient’s abdominal pain was relieved on the day of admission. Given the confirmed diagnosis of recurrent choledocholithiasis, both LCBDE and endoscopic retrograde cholangiopancreatography (ERCP) were considered feasible and appropriate therapeutic options. However, the patient presented with multiple large CBD stones, and our institution has limited clinical experience in large stone removal via ERCP. Due to these factors, we tended to prefer repeat LCBDE rather than ERCP. After detailed discussion with the patient and obtaining informed consent, repeated LCBDE was selected as the definitive intervention. Reoperation with T-tube reinsertion was performed on hospital day 2. Intraoperatively, all biliary stones were completely cleared. Postoperatively, a Hem-o-lok clip (red arrow) and a titanium clip (yellow arrow) were found to be embedded inside the stones (Figures 1C–E).

**Figure 2:**
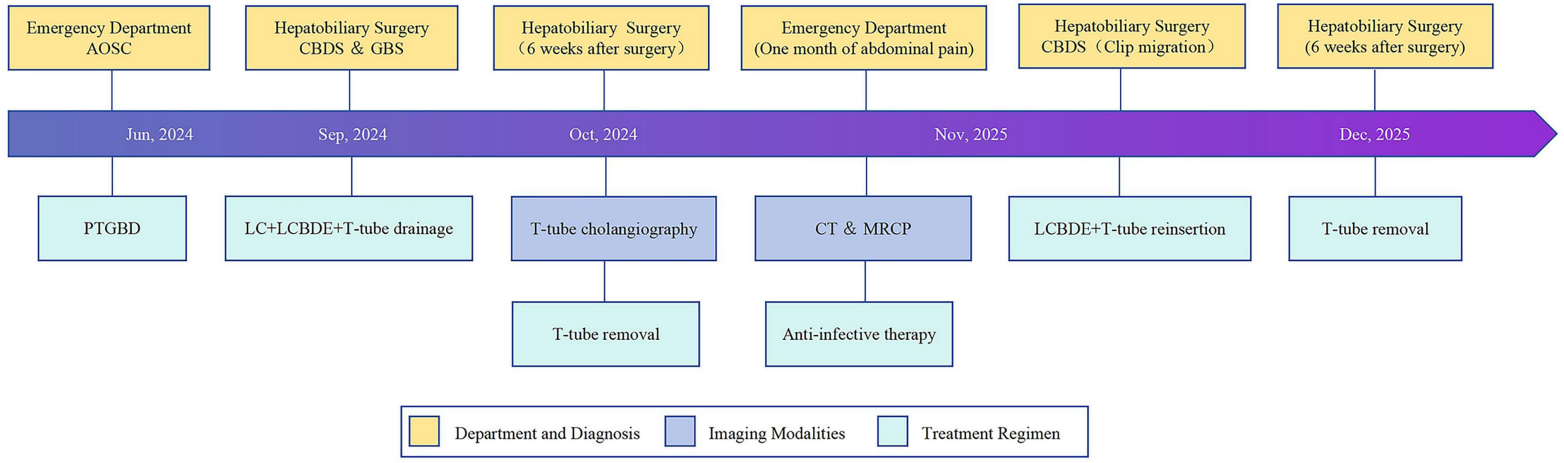
Clinical course timeline. July 2024: PTGBD for AOSC. September 2024: LC + LCBDE + T-tube drainage. October 2024: T-tube removal (normal cholangiography). November 2025: Readmission for abdominal pain; CT/MRCP showed clip migration and recurrent stones. Repeat LCBDE + T-tube drainage was performed. December 2025: T-tube removed (normal cholangiography). No recurrence to date. AOSC: acute obstructive suppurative cholangitis, LCBDE + T-tube: laparoscopic common bile duct exploration with T-tube placement, PTGBD: percutaneous transhepatic gallbladder drainage, CBDS: common bile duct stones, GBS: gallbladder stones, CT: computed tomography, MRCP: magnetic resonance cholangiopancreatography.

Postoperatively, the patient received ursodeoxycholic acid (UDCA), 500 mg orally once daily (qn), to prevent recurrent stone formation, and had an uneventful postoperative recovery. The T-tube was removed one month after surgery. At the 3-month follow-up, the patient remained in good general condition without obvious discomfort or adverse events. The clinical timeline covering medical history, surgical procedures and follow-up outcomes is presented in Figure 2.

## Discussion

Recurrent choledocholithiasis after laparoscopic biliary surgery manifests as recurrent biliary tract infections, abnormal liver function, and falsely elevated CA 19–9 levels, which should be differentiated from malignant biliary tract tumors. Recurrent choledocholithiasis is a common and intractable postoperative adverse event, affected by a combination of anatomical, pathophysiological, metabolic, and surgery-related factors (2–4). In terms of surgical factors, incomplete intraoperative stone clearance (10), unreasonable biliary tract reconstruction (11), and postoperative biliary stricture induced by surgical trauma (12) are the predominant iatrogenic causes of stone recurrence. In addition to the aforementioned conventional etiological factors associated with bile stasis and surgical procedures, rare iatrogenic postoperative adverse events can also trigger recurrent choledocholithiasis, among which surgical clip migration into the biliary tract is one of the most atypical and easily neglected causes.

Surgical ligation clips have greatly enhanced the efficiency of laparoscopic biliary surgery, and have now become the most widely used surgical consumable in laparoscopic procedures. Nevertheless, existing clinical reports indicate that these clips may migrate to other aberrant anatomical sites and regions. Postoperative clip migration, although rare, sometimes carries life-threatening risks (13). When surgical clips migrate, they may become lodged in the CBD, obstruct biliary drainage, and ultimately act as a nidus for stone formation (7). Severe cases can progress to acute suppurative cholangitis and even septic shock. Given the potential severity of this adverse event, clip migration into the CBD after laparoscopic biliary surgery warrants heightened attention from hepatobiliary surgeons.

To investigate clip migration after biliary surgery, we conducted a literature review aiming to clarify the potential underlying mechanisms of this complication and summarize corresponding clinical countermeasures. We systematically searched the MEDLINE, PubMed, Web of Science, and Google Scholar databases for relevant publications from January 2005 to April 2026. The search terms included “clip migration,” “laparoscopic common bile duct exploration”, “LCBDE”, “T-tube”, “biliary complication.” We included English-language clinical case reports, retrospective studies, reviews and letters, and excluded conference abstracts and duplicate publications. Duplicate cases retrieved from different databases were manually screened and excluded. All cases underwent rigorous screening: cases involving clip migration after LCBDE were included, while cases of clip migration after LC were excluded. Ultimately, 13 eligible studies were enrolled, comprising a total of 36 clinical cases (including the present case) (Table 1). To the best of our knowledge, there have been no published dual-clip migration cases after LCBDE with T-tube drainage.

**Table 1:** Summary of clinical data of 36 cases of clip migration after LCBDE.

**Ref.**	**Case No.**	**Age/Gender**	**T tube** **(Y/N)**	**Postoperative time**	**Examinations**	**Migration position**	**Clip type**	**Treatment**
Wang et al. (2009) (14)	3	42–73/ NA	Yes	1 month	Choledochoscopy	Unknown	Metallic Clip	Observation
					Choledochoscopy	T-tube sinus wall	Metallic Clip	Removed by choledochoscopy
					Choledochoscopy	T-tube sinus wall	Metallic Clip	Observation
Liu et al. (2012) (15)	8	35–76/2 Female, 6 Male	Yes	2–3 months	Cholangiography	CBD	Hem-o-lok	Removed by choledochoscopy
Qu et al. (2017) (16)	1	54/ Female	No	13 months	MRCP	CBD	Hem-o-lok	ERCP+EST
Zheng et al. (2018) (17)	6	NA/ Male	Yes	4 months	EGD	Duodenum wall	Hem-o-lok	Observation
		NA/ NA	No	50 months	NA	CBD	Hem-o-lok	CBDE (laparotomy operation)
		NA/ NA	No	27 months	NA	CBD	Hem-o-lok	LCBDE
		NA/ NA	Yes	6 weeks	Choledochoscopy	CBD wall	Absorbable clip	Removed by choledochoscopy
		NA/ NA	Yes	6 weeks	Choledochoscopy	CBD	Absorbable clip	Removed by choledochoscopy
		NA/ NA	Yes	6 weeks	Choledochoscopy	CBD	Absorbable clip	Removed by choledochoscopy
Pang et al. (2019) (13)	5	31/ Female	Yes	4 months	CT	T-tube sinus tract	Hem-o-lok	Removed by choledochoscopy
		60/ Female	Yes	3 months	CT	T-tube sinus tract	Hem-o-lok	Removed by choledochoscopy
		83/ Female	Yes	6 months	CT	T-tube sinus tract	Hem-o-lok	Removed by choledochoscopy
		72/ Female	Yes	6 months	CT	CBD	Hem-o-lok	CBDE (laparotomy operation)
		64/ Female	Yes	2 months	Cholangiography	CBD	Hem-o-lok	ERCP
Kou et al. (2019) (18)	1	84/ Male	Yes	36 months	CT, ERCP	CBD	Hem-o-lok	LCBDE (T tube)
Jiang et al. (2021) (19)	1	67/ Male	Yes	12 months	CT	CBD	Hem-o-lok	LCBDE (primary closure)
Tan et al. (2021) (20)	1	72/ Female	Yes	96 months	Ultrasound, MRCP	CBD	Hem-o-lok	LCBDE (T tube)
Jang et al. (2021) (21)	1	66/ Male	Yes	48 months	CT	CBD	Hem-o-lok	ERCP
Liu et al. (2022) (8)	1	59/ Female	Yes	2 months	CT, ERCP	CBD	Hem-o-lok	ERCP
Wu et al. (2023) (7)	4	62/ Female	Yes	19 months	MRCP	CBD	Hem-o-lok	LCBDE
		72/ Female	Yes	24 months	MRCP	CBD	Hem-o-lok	LCBDE
		69/ Male	Yes	17 months	MRCP	CBD	Hem-o-lok	LCBDE
		88/ Male	Yes	19 months	CT	CBD	Hem-o-lok	LCBDE
Huang et al. (2025) (22)	2	68/ Female	Yes	17 months	CT, MRCP	CBD	Hem-o-lok	ERCP+EST + ENBD
		74/ Male	Yes	9 weeks	CT	T-tube sinus tract	Hem-o-lok	Removed by choledochoscopy
Wu et al. (2026) (23)	1	76/ Female	Yes	36 months	CT, MRCP	CBD	Hem-o-lok	LCBDE (T tube)
Our case	1	75/ Male	Yes	16 months	CT, MRCP	CBD	Hem-o-lok and Metallic Clip	LCBDE (T tube)

CBD: common bile duct, MRCP: magnetic resonance cholangiopancreatography, CT: computed tomography, ERCP: endoscopic retrograde cholangiopancreatography, EST: endoscopic sphincterotomy, ENBD: endoscopic nasobiliary drainage, LCBDE: laparoscopic common bile duct exploration, EDG: esophagogastroduodenoscopy.

We further collected and summarized preoperative imaging findings and related diagnostic findings, and evaluated the clinical performance of different preoperative detection modalities in the diagnosis of clip migration (Supplementary Table S1). In our included cohort, only two patients received a correct diagnosis of clip migration via magnetic resonance cholangiopancreatography (MRCP). This accounts for only 25.0% of all patients that underwent MRCP examination. The primary underlying reason is that a migrated surgical clip within the CBD can readily act as a nidus for secondary stone formation. Once the surgical clip is completely encased by newly formed stones, imaging can only reveal the overall outline of the CBD stones, making it impossible to accurately distinguish between the stone and the migrated clip. Similarly, both T-tube cholangiography and ERCP depend on cholangiographic visualization to obtain diagnostic results. As a result, when the migrated surgical clip is completely embedded inside the calculus, these imaging modalities also fail to detect the clip migration. By contrast, CT scanning can directly identify migrated surgical clips, as it clearly distinguishes variations in tissue density and morphological differences. In the present case series, CT achieved a notably high diagnostic rate of 92.3% (12/13) by directly visualizing characteristic high-density clip shadows. This finding indicates that CT may be regarded as the first-line imaging modality for early detection of clip migration after biliary surgery. However, considering the potential selection bias associated with imaging examinations, CT diagnostic rates may overestimate the true real-world sensitivity, and accumulation of more clinical cases will help us more clearly define the diagnostic value of CT in detecting clip migration.

To date, there is still no unified clinical consensus on the exact pathogenic mechanism of clip migration after laparoscopic biliary surgery. Previous clinical observations have indicated that clip migration after LC isprimarily attributable to a variety of perioperative factors and local tissue-related factors. Firstly, persistent chronic local inflammation gradually leads to progressive erosion of the peribiliary tissue and the cystic duct wall. This process ultimately results in tissue defects, causing the surgical clip to gradually migrate into the lumen of the CBD (20). Secondly, the chronic inflammatory response and foreign body rejection reaction caused by the surgical clip can also damage the bile duct wall, ultimately leading to clip migration (20). Thirdly, inappropriate surgical clip application, including improper placement or misuse of Hem-o-lok clips, may initiate a cascade of adverse events: incomplete occlusion of the cystic duct subsequently leads to postoperative bile leakage and local cholesteatoma formation, which further aggravates peribiliary inflammatory adhesion and ultimately promotes clip loosening and migration (8). Fourthly, the anatomical structure of the cystic duct-CBD junction is naturally flared and structurally unstable; if the surgical clip is placed excessively close to the biliary tract during ligation, it may gradually retract inward into the CBD under continuous surrounding tissue pressure after the loss of gallbladder traction (20).

Specifically for patients undergoing LCBDE, several additional procedural risk factors may contribute to postoperative clip migration. Our pooled literature review found the following observations among the 36 reported clip migration cases: T-tubes were placed intraoperatively in 33 patients (91.7%), and migration predominantly occurred in the CBD (77.8%) and the T-tube sinus tract (16.7%) (Supplementary Table S2). During choledochoscopy performed immediately after T-tube removal, migrated surgical clips were detected in the T-tube sinus tract among multiple patients (13, 14, 22). In some severe cases, the surgical clips had even migrated completely into the CBD (15, 17). Upon reviewing the CT images obtained before T-tube removal, we identified two high-density foreign bodies adjacent to the T-tube entry site of the CBD. These findings indicate that T-tube placement may be a potential risk factor for clip migration after LCBDE.

Preoperative CT scan revealed that the two Hem-o-lok clips placed on the cystic duct remained in their original position (Supplementary Figure S1). Based on these imaging findings, we hypothesize that the surgical clips placed on the cystic artery may be more prone to loosening. Given that these loose clips are anatomically proximal to the CBD and the T-tube, they may possibly become gradually embedded in the T-tube sinus tract wall. Once the T-tube has been removed, the sinus tract loses its mechanical structural support. In particular, excessive dissection of pericholedochal soft tissue further weakens the local tissue stability; we hypothesize that persistent fluctuations in intra-abdominal pressure may ultimately drive clip migration into either the sinus tract or the CBD. The potential pathogenic mechanisms underlying clip migration after LCBDE differ substantially from those after LC, particularly in patients undergoing LCBDE combined with T-tube drainage. Studies have demonstrated that absorbable clips can reduce the risk of postoperative complications such as inflammation and biliary fistula compared with conventional surgical clips, thereby lowering the incidence of clip migration (24). Clip-free techniques, reduced use of metallic clips, or application of energy devices can effectively avoid long-term clip-associated complications (25). In addition, primary biliary suture is safe and effective for specific patient populations (26), avoiding T-tube removal and maintaining a stable peribiliary microenvironment can also help reduce the likelihood of clip migration.

Such procedural discrepancies indicate that the majority of clip migration events after LCBDE may be preventable with standardized surgical manipulation. Therefore, we recommend minimizing the overuse of non-absorbable ligation materials, including Hem-o-lok and metallic clips, and adhering to the following surgical precautionary principles: 1. The cystic artery should be ligated close to its origin, avoiding proximity to the CBD incision. 2. Reserve a residual cystic duct of at least 0.5 cm in length when the duct is in a relaxed state; 3. All surgical clips should be placed sufficiently distant from the CBD incision; 4. Excessive skeletonization and dissection of pericholedochal soft tissue should be strictly avoided; 5. Primary CBD suture closure is preferred whenever intraoperative conditions permit; 6. Routine intraoperative choledochoscopy is strongly advised prior to T-tube removal. Nevertheless, the above standardized surgical protocols cannot completely eliminate the risk of clip migration, as persistent local inflammation secondary to residual biliary infection, bile fistula, and other postoperative complications may still induce clip migration. Notably, clip migration has been sporadically reported even after the application of absorbable clips (17), and this adverse event also remains an occasional possibility despite successful primary CBD closure (16, 17).

Different clinical presentations require targeted and differentiated therapeutic strategies. For surgical clips migrated into the CBD, both LCBDE and ERCP combined with EST are considered viable and effective therapeutic alternatives. For patients with an indwelling T-tube, intraoperative choledochoscopy represents the optimal approach for retrieving migrated surgical clips located within the CBD or T-tube sinus tract (13, 17). For surgical clips migrated into the digestive tract wall, conservative management with close long-term follow-up can be adopted (25). Although such embedded clips may spontaneously detach and be excreted via the intestinal tract, continuous clinical vigilance is mandatory to prevent the potential risk of gastrointestinal perforation. Given that reports of clip migration remain relatively limited, the accumulation of more clinical cases may help focus attention on technical details of LCBDE surgical procedures to reduce the incidence of postoperative clip migration.

The strength of the present case lies in its focus on clip migration after LCBDE with T-tube drainage, which will prompt surgeons to pay more attention to the details of T-tube placement and removal, as well as the precautions that should be taken during the procedure. Given that reports of clip migration remain relatively limited, the accumulation of more clinical cases may help focus attention on technical details of LCBDE surgical procedures to reduce the incidence of postoperative clip migration. Furthermore, as the follow-up period in this study was relatively short, it was not possible to assess the risk of other long-term complications or recurrence of CBD stones postoperatively.

## Conclusion

Clip migration into the CBD following LCBDE is an uncommon iatrogenic complication; however, it can lead to life-threatening biliary complications and poor clinical outcomes. The T-tube sinus tract acts as a potential anatomical passage for clip displacement, and a migrated surgical clip inevitably serves as the nidus for recurrent choledocholithiasis. To effectively reduce the incidence of this rare complication, hepatobiliary surgeons must exercise meticulous caution during clip ligation, minimize the overuse of non-absorbable titanium and Hem-o-lok clips, maintain an appropriate length of the cystic duct stump, ensure surgical clips are fully closed, and avoid excessive pericholedochal skeletonization. For patients requiring mandatory T-tube drainage, routine choledochoscopy at the time of T-tube removal is recommended to enable early detection and timely clip retrieval. Overall, sustained high clinical vigilance, together with prompt surgical or endoscopic intervention, is essential to achieve optimal long-term prognosis in patients with this unusual biliary complication.

## Patient perspective

Last year, I was diagnosed with cholecystolithiasis and choledocholithiasis, and underwent simultaneous laparoscopic cholecystectomy (LC) and laparoscopic common bile duct exploration (LCBDE) with T-tube drainage. The procedure went smoothly, and my postoperative recovery was uneventful. The T-tube was removed successfully without any complications, and over the following year I maintained a normal diet with no discomfort such as abdominal pain or fever. However, for the past month I have experienced recurring abdominal pain. The frequent episodes frustrated me greatly, until this new surgery completely resolved my troubles. I am very grateful to my doctor for his support and guidance during this difficult time. I am now fully recovering, and have had no more episodes of abdominal pain, for which I am truly grateful.

## Data Availability

The original contributions presented in the study are included in the article/Supplementary material, further inquiries can be directed to the corresponding author/s.
