# Supplementary material for: Case Report: Clip migration induced recurrent choledocholithiasis after laparoscopic common bile duct exploration
Source: Front Med (Lausanne). 2026 Jun 3;13:1869401. doi: 10.3389/fmed.2026.1869401 (PMC13271967; doi:10.3389/fmed.2026.1869401)
Supplement: Supplementary file 1 [file Data_Sheet_1.DOCX]

Supplementary Material

# **Supplementary Tables**

# **Table S1. Diagnostic capacity of preoperative examination**

| **Preoperative examination** | **Found clips [n (%)]** | **Unfound clips [n (%)]** |
| --- | --- | --- |
| CT | 12 (92.3) | 1 (7.7) |
| MRCP | 2 (25.0) | 6 (75.0) |
| Choledochoscopy via T-tube sinus tract | 6 (100) | 0 |
| Cholangiography via T-tube | 1 (11.1) | 8 (88.9) |
| ERCP | 1 (50) | 1 (50) |
| Ultrasound | 0 | 1 (100) |
| EGD | 1 (100) | 0 |

MRCP: magnetic resonance cholangiopancreatography, CT: computed tomography, ERCP: endoscopic retrograde cholangiopancreatography, EDG: esophagogastroduodenoscopy

# **Table S2. Summary of initial operation, migration position and treatment**

|  | **n (%)** |
| --- | --- |
| Initial operation |  |
| LCBDE with T tube drainage | 33 (91.7) |
| LCBDE with primary closure | 3 (8.3) |
| Migration position |  |
| CBD | 28 (77.8) |
| T-tube sinus tract | 6 (16.7) |
| Duodenum | 1 (2.8) |
| Unknown | 1 (2.8) |
| Treatment |  |
| LCBDE or laparotomy operation | 12 (33.3) |
| ERCP | 5 (13.9) |
| Removed by choledochoscopy | 16 (44.4) |
| Observation | 3 (8.3) |

CBD: common bile duct, ERCP: endoscopic retrograde cholangiopancreatography, LCBDE: laparoscopic common bile duct exploration

# **Supplementary Figures**


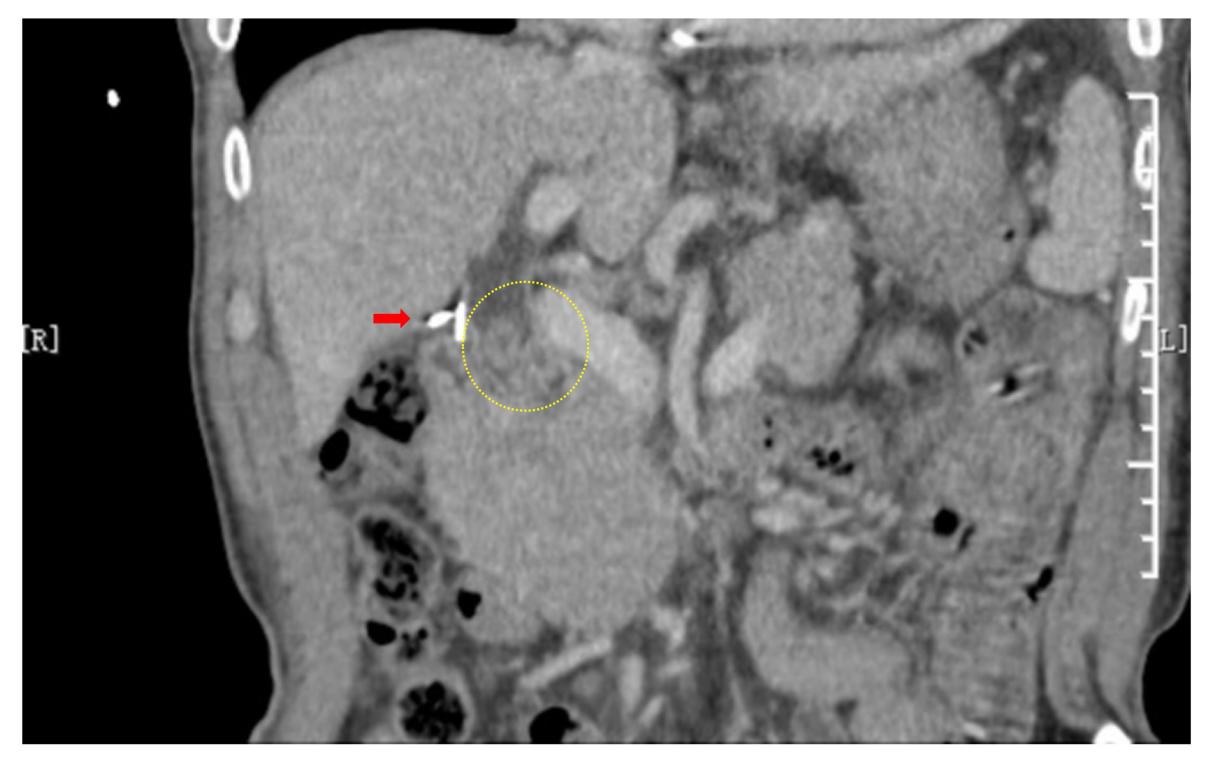


**Supplementary Figure 1.** CT scan revealed that two surgical clips on the cystic duct were still in place (red arrows), with CBD dilation accompanied by stone formation (yellow ellipse).
